# A Novel Optimal Joint Resource Allocation Method in Cooperative Multicarrier Networks: Theory and Practice

**DOI:** 10.3390/s16040522

**Published:** 2016-04-12

**Authors:** Yuan Gao, Weigui Zhou, Hong Ao, Jian Chu, Quan Zhou, Bo Zhou, Kang Wang, Yi Li, Peng Xue

**Affiliations:** 1 Xichang Satellite Launch Center, Xichang 615000, China; wgzhou@nudt.edu.cn (W.Z.); aohong76@aliyun.com (H.A.); chujian_sat@tom.com (J.C.); zhouquanxslc@foxmail.com (Q.Z.); zhoubowin78@sina.com (B.Z.); wangkangdream@gmail.com (K.W.); 2 State Key Laboratory on Microwave and Digital Communications, National Laboratory for Information Science and Technology, Tsinghua University, Beijing 100084, China; liyi@rdfz.cn; 3 China Defense Science and Technology Information Center, Beijing 100030, China; 4 The High School Affiliated to Renmin University of China, Beijing 100080, China; 5 Naval Aeronautical and Astronautical University, Yantai 264000, China

**Keywords:** distributed compression, backhaul optimization, user pairing, uplink CoMP, virtual MIMO

## Abstract

With the increasing demands for better transmission speed and robust quality of service (QoS), the capacity constrained backhaul gradually becomes a bottleneck in cooperative wireless networks, e.g., in the Internet of Things (IoT) scenario in joint processing mode of LTE-Advanced Pro. This paper focuses on resource allocation within capacity constrained backhaul in uplink cooperative wireless networks, where two base stations (BSs) equipped with single antennae serve multiple single-antennae users via multi-carrier transmission mode. In this work, we propose a novel cooperative transmission scheme based on compress-and-forward with user pairing to solve the joint mixed integer programming problem. To maximize the system capacity under the limited backhaul, we formulate the joint optimization problem of user sorting, subcarrier mapping and backhaul resource sharing among different pairs (subcarriers for users). A novel robust and efficient centralized algorithm based on alternating optimization strategy and perfect mapping is proposed. Simulations show that our novel method can improve the system capacity significantly under the constraint of the backhaul resource compared with the blind alternatives.

## 1. Introduction

In LTE-Advanced Pro related systems, due to the spectrum resource limitations, the system capacity cannot increase without limitation, so cooperative transmission has been adopted to enhance transmission speed, especially for cell-edge users with poor performance. However, the link to transmit cooperative information is also capacity constrained when the amount of cooperative data is huge, so it will also face a bottleneck. Such conditions will get worse with the quick development of the Internet of Things (IoT), as the number of terminals working in cooperative mode will deteriorate the wireless environment. To tackle this problem, various inter-cell interference mitigation techniques have been adopted. One typical method is Coordinated Multipoint Transmission/Reception (CoMP) [1]. This operation avoids or exploits interference through coordination among different base stations, which is implemented by information sharing via backhaul among coordinated BSs. Generally, there are two ways to implement coordinated transmission based on different types of cooperation: (1) coordinated beamforming or coordinated scheduling (CB/CS), where only neighbor channel state information (CSI) is exchanged for coordinated scheduling and beam forming [2,3]; (2) Joint Processing (JP): Both data and CSI are shared using cooperative links for joint decoding or transmission [4,5], which inevitably brings increased pressure on the system backhaul. The problem of backhaul resource constraints in realistic systems is more serious, particularly when full cooperation based on data sharing is employed, especially in multi user scenario [6].

In uplink transmission, a multi-user decoding scheme is proposed to increase system performance. Joint decoding means the signals received at different base stations (BSs) are jointly decoded through exchanging quantized information among cooperative ones [7,8]. This scheme can exploit diversity and cooperative gain to a large extent, similarly to virtual MIMO (Multiple-Input Multiple-Output) [9], which provides extra overhead to the capacity-limited backhaul. Therefore, it is necessary to reduce the redundancy in shared information transmitted through backhaul links and improve the efficiency of backhaul utilization.

In this case, the problems could be solved using distributed source coding with side information, which is commonly used by researchers [10,11]. Based on this idea, a new backhaul efficient approach method called distributed compression is proposed in [12,13]. In this method, multiple cooperative BSs compress and forward their received signals through backhaul to the central BS for joint decoding. As these compressed versions carry only necessary information for the central BS, backhaul overhead is greatly reduced and joint processing gain is obtained at the same time. It was shown in [14] that this compress-and-forward scheme achieves good performance compared with the methods without such operations. However, this work is based on a single carrier network. The optimal backhaul resource allocation in more practical multicarrier systems has not been considered yet.

In multicarrier systems, the problems become more complex due to the increased dimensions, so researchers are trying to solve such problems by adding more constraints or weakening the scenario. In [15,16,17,18], a capacity constrained backhaul transmission environment has been adopted in wireless networks, and methods using Markov Control are given to adopt the optimal policy, but the constraint is strict and the optimal solution could only be obtained offline. In [19,20,21], the authors try to rewrite the problem as a continuous optimal power allocation policy under the system capacity constraint. In this way, the discrete optimization problem has been relaxed to a continuous scenario and the bandwidth resource allocation in backhaul links is not mentioned. In [22,23,24,25], the scenario is limited to uplink multiple access, and the authors study the resource allocation (e.g., transmit power, bandwidth and number of antennas) in uplink transmissions under a fixed backhaul bandwidth, but the arrangement of users and the optimal backhaul resource allocation are not mentioned. In [26], a smart grid scenario has been considered to solve the optimal problem, where big data related methods are adopted, but there is little innovation in solving such an integer programming method, and the solution rather relies on the advantages of distributed computing architecture. In [27,28,29], the problem of uplink CoMP with imperfect CSI under capacity limited backhaul is studied, but the solutions are mainly about the uplink resource allocation and decoding design, not the backhaul link allocation. In [23], the authors discuss the problem in a centralized computing scenario, so backhaul links become transmission links, and the cooperation consumption will cost no more resources, so such problems could be easily solved using convex optimization tools.

In this work, we are trying to figure out the optimal solution of the uplink multiuser CoMP with limited backhaul resources using convex optimization tools. The rest of this paper is organized as follows: In Section 2, we summarize the contributions of this paper. In Section 3 and Section 4, the system model and problem formulation are given. After that, the proposed novel algorithm is introduced in Section 5. Finally, numerical results including theoretical and system level simulation results are presented in Section 6 before conclusions are drawn.

## 2. Main Contributions of This Work

In this paper, we formulate a joint 3-dimension optimization problem of user pairing, subcarrier mapping and backhaul resource sharing between different pairs (subcarriers). A low complexity but efficient algorithm based on alternating optimization strategy and perfect mapping is proposed to solve this mixed integer programming problem. Simulations show that this allocation algorithm can improve the system capacity significantly compared with the blind alternatives. The contributions of this manuscript can be summarized as follows:

1. In this work, we divide the complex integer programming problem into three steps: The user pairing, resource allocation and compression of the system noise. This operation helps reduce the dimensionality and the complexity of the algorithm without losing system performance significantly. Related results of this work could be easily extended to the IoT scenario;
2. We have proved the optimal result of the joint optimization problem using the three dimension optimization approach, and the theoretical results indicate that our proposed method could solve the multi-steps optimization problem at a satisfactory speed, so the system performance is improved significantly compared to reference methods;
3. To evaluate the availability of our proposed method, we first propose a system level simulation to check the effectiveness of our method. Results indicate that our given method could be used in real systems and the performance meets the expectations studied in the theoretical analysis.

## 3. System Model Description

**Notation:** In the following, boldface lowercase letters are used to denote vectors whereas boldface uppercase letterss denote matrices. ${\left(\cdot \right)}^{T}$ and ${\left(\cdot \right)}^{†}$denote the transpose and conjugate transpose of their matrix arguments, respectively. I(x;y) denotes the mutual information function between x and y. $R_{+}$ denotes the nonnegative real domain. $I_{n}$ denotes the n × n unitary matrix.

### 3.1. Preliminary

**Figure 1:**
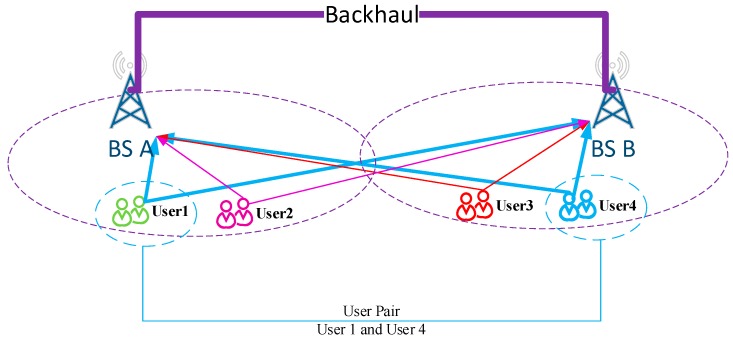
Cooperative Scenario with capacity constraint backhaul system. Users located at different base stations will transmit uplink information and base stations will receive information from every users.

First, we give a brief introduction to the compress-and-forward scheme [13] in two BSs case (depicted as Figure 1). BS A acts as central node to execute joint decoding. BS B acts as cooperative node, forwarding its information via an unidirectional backhaul link from BS B to BS A. The specific operations are as below:

BS B compresses and encodes its received signal waiting to be transmitted via a backhaul link, which is a mapping from a received sequence ${\left\{y_{B}^{n}\right\}}_{n=1}^{N_{s}}\in Y_{B}^{N_{s}}$ to a codeword of length $N_{s}$, $r_{BH}$ is the compression rate:

(1) $$f:Y_{B}^{N_{s}}\to \{1,2,\ldots ,2^{N_{s}r_{BH}}\}$$

BS A reconstructs the compressed signal with its received signal ${\left\{y_{A}^{n}\right\}}_{n=1}^{N_{s}}\in Y_{A}^{N_{s}}$ as side information. It could be expressed using a mapping function:

(2) $$g:\{1,2,\ldots ,2^{N_{s}r_{BH}}\}\times Y_{A}^{N_{s}}\to {\hat{Y}}_{B}^{N_{s}}$$

Then BS A jointly decodes users’ information from $\left(y_{A}^{N_{s}},{\hat{y}}_{B}^{N_{s}}\right)$.

This compression and reconstruction process has been modeled as a Gaussian test channel:

(3) $${\hat{y}}_{B}=y_{B}+q\;q\sim NC(0,\eta )$$

where *q* is referred to as compression noise, with variance η. Under this model, the optimum compression code parameterized by compressed noise is designed based on maximizing the sum rate criteria in [13]. We will verify, later in the paper, that it is also applicable in more widely used multicarrier systems.

In the following part, we will give the modeling of our proposed scenario using the notations, equations and models presented above.

### 3.2. Signal and System Model

The system model is depicted in Figure 1. It consists of two single-antenna BSs and *N* single-antenna users designated to each (*N* equals 4 in this figure, and it is not limited to 4 in real scenarios). These users are paired and mapped onto *K* subcarriers for uplink transmission. For example, User 1 located at base station A is paired with User 4 in base station B and the two users are mapped on the subcarrier denoted by a blue line. In this model, backhaul resource is defined *C_BH_* bits per channel use (bpcu). It means cooperation between BS A and BS B is under the constraint of *C_BH_* bits for lossless transmission.

The channel model in frequency domain is illustrated as follows:

(4) $$y_{A}=H_{A}s+n_{A}$$

(5) $$y_{B}=H_{B}s+n_{B}$$

$y_{A}(y_{B})\sim C^{K\times 1}$ is the received signal of BS A (or BS B), $s\in C^{2K\times 1}$ is the transmitted symbol vector with the k−th element, $s_{k}$ is denoted as user information modulated on subcarrier k. $H_{A(B)}=diag(h_{A(B),k})\in C^{K\times 2K}$ describes the channel matrix, where the diagonal element $h_{A,k}$($h_{B,k}$) is the channel response from users mapped on subcarrier k to BS A (or BS B). The noise vector $n_{A}(n_{B})\in C^{K\times 1}$ is a realization of a zero-mean circularly symmetric complex Gaussian random process: $n_{A}(n_{B})\sim NC(0,\sigma^{2}I_{K})$, where $\sigma^{2}$ is noise variance.

The compression and decompression process is similar to the scalar case:

(6) $${\hat{y}}_{B}=y_{B}+q\;q\sim NC(0,\Phi )$$

$q\in C^{K\times 1}$is the compression noise vector with zero mean and $\Phi \in C^{K\times K}$ is covariance variance matrix of compression noise. In the following part, the modeling and formulation will be expressed.

## 4. Joint Optimization Problem Formulation

### 4.1. The Achievble Rate

Assume different subcarriers are perfectly orthogonal to each other [18,20,22], the achievable sum rates should satisfy:

(7) $$f\leq I(s;y_{A}{\hat{y}}_{B})=\sum_{k=1}^{K}I(s_{k};y_{A,k}{\hat{y}}_{B,k})$$

where $y_{A,k}$ is the signal received by BS A on the subcarrier k. ${\hat{y}}_{B,k}$ is the reconstructed signal decompressed by BS A. (BS A is the central node to process joint decoding as defined above). 

### 4.2. The Backhaul Overhead

(8) $$R_{BH}=I({\hat{y}}_{B};y_{B})=\sum_{k=1}^{K}I({\hat{y}}_{B,k};y_{B,k})\leq C_{BH}$$

Let $R=\{r|r\in R_{+}^{K},1^{T}r\leq C_{BH}\}$ denotes the feasible set of all possible compression rates vector, where the *k*-th element *r_k_* denoted as the compression rate used on the *k*-th subcarriers:

(9) $$r_{k}=I({\hat{y}}_{B,k};y_{B,k})$$

Obviously, this rate vector can also be viewed as a backhaul resource allocation vector. We will use these two terms without distinction in the rest of the paper.

### 4.3. Joint Optimization Problem

The objective of our work is maximizing the system throughput achieved by cooperation. Therefore, we formulate the joint optimization problem by combining backhaul resource allocation with user pairing and subcarrier mapping. We introduce a set of binary variables $x_{i,j,k}\in \{0,1\}$, which represent the mapping status of all user sets. When $x_{i,j,k}=1$, it means the *i*-th user in cell A is paired with the *j*-th user in cell B and they are both mapped on subcarrier *k*. Otherwise, $x_{i,j,k}=0$. Recalling Equations (7) and (8), both sum rate and backhaul overhead must satisfy subcarrier additivity. This is guaranteed by the independency of subcarriers. Substituting Equation (9) into Equation (8), and combining variable $x_{i,j,k}$, we setup an optimization problem aimed at maximizing system throughput with limited backhaul resource constraints:

(10) $$P1:\;\underset{x,r,\Phi }{\text{max}}\;\sum_{i=1}^{N}\sum_{j=1}^{N}\sum_{k=1}^{K}x_{i,j,k}f_{i,j,k}$$

(11) $$s.t.\;\sum_{j=1}^{N}\sum_{k=1}^{K}x_{i,j,k}=1,\;\forall i$$

(12) $$\sum_{i=1}^{N}\sum_{k=1}^{K}x_{i,j,k}=1,\;\forall j$$

(13) $$\sum_{i=1}^{N}\sum_{j=1}^{N}x_{i,j,k}\leq 1,\;\forall k$$

(14) $$r^{T}1\leq C_{BH}\;$$

$f_{i,j,k}$ is the achievable rate of user pair (i,j) who occupy the subcarrier k. It is a function of user pairing, subcarrier mapping and backhaul resource allocated to that subcarrier. Equations (11)–(13) are assignment constraints. For convenience, we denote the objective function as:

(15) $$F(x,r,\Phi )=\sum_{i=1}^{N}\sum_{j=1}^{N}\sum_{k=1}^{K}x_{i,j,k}f_{i,j,k}$$

It is clear the optimization problem given in Equations (10)–(14) is a complex optimization problem and could not be solved in polynomial time, so in the following part, our proposed 3-step sub-optimal solution is given.

## 5. Our Proposed Novel Resource Allocation Scheme

As Equations (10)–(14) represent a nonlinear mixed integer programming, it is difficult for us to tackle directly. According to [14], we can maximize some variables first and then maximize over the remaining variables. Therefore, to explore the internal features of our problem, we observe some variables taking others as fixed.

### 5.1. Opitmize Compression Noise Φ

For the fixed assignment variables $\underline{x}$ and backhaul resource allocation vector $\underline{r}$, let $F(\underline{x},\underline{r},\Phi )$ presents objective function. For any given $\left(x_{i,j,k},r_{k}\right)$, the signals received by BS A and BS B are:

(16) $$y_{A,k}=h_{i,A}s_{i}+h_{j,A}s_{j}+n_{A}$$

(17) $$y_{B,k}=h_{i,B}s_{i}+h_{j,B}s_{j}+n_{B}$$

Because of the assumption of orthogonality between subcarriers, Φ is a diagonal matrix, *i.e.*, $\Phi =diag(q_{k})$, $q_{k}\sim NC(0,\eta_{k})$ is the compression noise of compression code used by subcarrier *k*:

(18) $${\hat{y}}_{B,k}=y_{B,k}+q_{k}$$

The sum rate of user pair *i* and user *j* on subcarrier *k* is:

(19) $$f_{i,j,k}=I(s_{i}s_{j};y_{A,k}{\hat{y}}_{B,k})$$

And the sub-problem of maximizing achievable pair rate under given fixed backhaul rate is:

(20) $$\begin{array}{ll} \underset{\eta_{k}}{\text{max}} & I(s_{i}s_{j};y_{A,k}{\hat{y}}_{B,k})\\ s.t. & I(y_{A,k};{\hat{y}}_{B,k})\leq r_{k} \end{array}$$

The optimal solution $q_{k}$ to the above problem is given in [13]:

(21) $$q_{k}^{*}=e_{i,j,k}/(2^{r_{k}}-1)$$

where $e_{i,j,k}$ is the eigenvalue of conditional covariance associated with user *i* and user *j* on subcarrier *k*. Given the knowledge of $y_{A,k}$, $y_{B,k}$ is Gaussian distributed and the conditional covariance denoted as $R_{B|A}^{i,j,k}$. It can be computed as follows:

(22) $$R_{B|A}^{i,j,k}=R_{B}^{i,j,k}-R_{B,A}^{i,j,k}{\left(R_{A}^{i,j,k}\right)}^{-1}R_{A,B}^{i,j,k}$$

and:

(23) $$R_{A}^{i,j,k}=P(|h_{i,A}^{k}{|}^{2}+|h_{j,A}^{k}{|}^{2})+\sigma^{2}$$

(24) $$R_{A,B}^{i,j,k}=P(h_{i,A}^{k}h_{i,B}^{k*}+h_{j,A}^{k}h_{j,B}^{k*})$$

(25) $$R_{B}^{i,j,k}=P(|h_{i,B}^{k}{|}^{2}+|h_{j,B}^{k}{|}^{2})+\sigma^{2}$$

(26) $$R_{B,A}^{i,j,k}=P(h_{i,B}^{k}h_{i,A}^{k*}+h_{j,B}^{k}h_{j,A}^{k*})$$

where $R_{A}^{i,j,k}$ and $R_{B}^{i,j,k}$ denote the covariance of the received signal on subcarrier *k* at BS A and BS B, while $R_{A,B}^{i,j,k}$($R_{B,A}^{i,j,k}$) denotes the cross-correlation between the BS A(B) and BS B(A) observations.

As $R_{B|A}^{i,j,k}$ is a scalar, the eigenvalue $e_{i,j,k}$ of conditional covariance equals to itself. Substituting Equation (21) into Equation (19), the optimized pair rate $f_{i,j,k}$ is:

(27) $$\begin{array}{cc} f_{i,j,k}^{*}= & \log_{2}\left(1+P(|h_{i,A}^{k}{|}^{2}+|h_{j,A}^{k}{|}^{2})/\sigma^{2}\right)\\ & +\log_{2}\left(e_{i,j,k}2^{r_{k}}/((2^{r_{k}}-1)\sigma^{2}+e_{i,j,k})\right) \end{array}$$

This equation indicates that the achievable rate of the pair can be decomposed into two parts. The first one is attributed to $y_{A,k}$, the second one is related to two factors: (1) the additional information offered by $y_{B,k}$; (2) the backhaul rate allocated to subcarrier *k*.

It can be easily verified that $f_{i,j,k}^{*}$ is a concave function in $r_{k}$. Based on above formulations, F(x,r,Φ) could be reduces to F(x,r,Φ(r)), which is now related to x and r.

### 5.2. Optimizing Backhaul Resource Allocation Vector r

For a fixed user pairing and subcarrier mapping $\underline{x}$, F(x,r,Φ(r)) is concave in r and constraint set Equation (14) is convex. The optimal backhaul resource allocation vector $r^{*}$ can be efficiently found by applying KKT conditions or CVX toolbox [30].

### 5.3. Optimizing User Pairing and Subcarrier Mapping x:

Given $\underline{r}$ and $\Phi (\underline{r})$, the objective function becomes:

(28) $$F(x,\underline{r},\Phi (\underline{r}))=\sum_{i=1}^{N}\sum_{j=1}^{N}\sum_{k=1}^{K}x_{i,j,k}R_{i,j,k}(x_{i,j,k})$$

This is a standard three dimensional assignment problem and is NP hard in most cases [31]. In order to deal with it practically, we have developed a sub-optimal method which also has pretty good performance. This principle of this method is to reduce this three-dimensional assignment problem to a two-dimensional assignment problem.

Similarly to Equation (27), the whole sum rate achieved by adding all subcarriers is composed of two terms: the first one is the sum rate when only $y_{A}$ is used for decoding, the second one is additional information obtained from BS B, which is limited by two factors. One is the additional information $y_{B}$ can offer conditioned by $y_{A}$, the other one is how many resources the backhaul link could afford. These two factors are described by the bound of multiple access and cut set bound in information theory [12]. Based on these observations above, it is reasonable to let $y_{A}$ be fulfilled with data as much as possible, so that the information required from BS B is less. This idea can also maximize the worst case, where the backhaul resource is close to zero.

Therefore, for each timeslot, we can first map scheduled *K* users in base station A onto the *K* subcarriers such that achievable sum rate of these users is maximized, which is also consistent with the no-cooperation scenario. The procedure can be easily achieved by selecting the best user for each subcarrier. Here, we use a mapping function α(i):{1,2,...,K}→{1,2,...,K} to denote this assignment. After that, the objective function is reduced to:

(29) $$F(x,\underline{r},\Phi (\underline{r}))=\sum_{i=1}^{N}\sum_{k=1}^{K}x_{i,j,\alpha (i)}R_{i,j,\alpha (i)}(x_{i,j,\alpha (i)})$$

This α(i) procedure is a suboptimal procedure but it reduces the dimensions of the problem expressed in Equation (28) from three to two. We can observe this pre-mapping brings about a tiny performance loss compared to the optimal solution.

Then we pair users in base station A with users in base station B by using the function β(i):{1,2,...,K}→{1,2,...,K}, which is a perfect matching problem [15]. By relaxing the binary variables to continuous variables in [01] and solving this linear programming problem, we get the exactly optimal binary solution.

### 5.4. Proposed Algorithm

Through the discussions presented in Section 5.1, Section 5.2, Section 5.3 and Section 5.4, we convert the intractable problem described by Equations (10)–(14) into the tractable suboptimal problem as below:

(30) $$P2:\underset{\beta ,r,\Phi }{\text{max}}\;\sum_{i=1}^{N}\sum_{j=1}^{N}\sum_{k=1}^{K}x_{i,j,k}f_{i,j,k}$$

(31) $$s.t.\;\sum_{j=1}^{N}\sum_{k=1}^{K}x_{i,j,k}=1,\;\forall i$$

(32) $$\sum_{i=1}^{N}\sum_{k=1}^{K}x_{i,j,k}=1,\;\forall j$$

(33) $$\sum_{i=1}^{N}\sum_{j=1}^{N}x_{i,j,k}\leq 1,\;\forall k$$

(34) $$r^{T}1\leq C_{BH}$$

(35) α(i)=k

(36) β(i)=j

In conclusion, for fixed x and r, optimal $\Phi^{*}$ can be explicitly expressed by r; for fixed x and Φ, optimal $r^{*}$ is obtained by solving a convex problem; for fixed Φ and r, x is decomposed into two variables α and β, where the former guarantees worse case optimality and the latter is linear relaxed without any optimality loss [31,32,33].

Therefore, we have developed an alternative optimization algorithm (Algorithm 1) by solving the convex problem and linear programming alternately. According to [16], this algorithm converges to the optimal solution of problem P2 defined by Equations (30)–(36). We summarize it in the following [17,18]:
**Algorithm 1:** The Proposed Novel Resource Allocation Algorithm1.Mapping users in cell A onto K subcarriers using α2.Initialize $\beta^{\left(0\right)}$ and $r^{\left(0\right)}$ randomly, let t=0.3.**Repeat:**4.t=t+15.Get $r^{\left(t\right)}$ by convex optimization;6.Compute $\Phi^{\left(t\right)}=diag(\eta_{1}^{\left(t\right)},\eta_{2}^{\left(t\right)},...,\eta_{K}^{\left(t\right)});$ using (18)7.Compute $f_{i,j,k}^{\left(t\right)}(\beta^{\left(t-1\right)},r^{\left(t\right)})$ using (24)8.Get $\beta^{\left(t\right)}$ by linear programming.9.Compute objective function $f^{\left(t\right)}$ given $r^{\left(t\right)},\Phi^{\left(t\right)},\beta^{\left(t\right)}$10.**Until** $F^{\left(t\right)}$ converges

In our proposed algorithm, there are three steps:

The first step is the optimization of compression noise, where the complexity is o(N × K)
The second step is resource allocation, where the complexity is: $o(length(\bar{x}))$
The last step is user pairing and subcarrier mapping, the complexity is: o(N! × K)

So the complexity of our method is: $length(\bar{x})\;\times \;o(N\;\times \;K)\;\times \;o(N!\;\times \;K)$

## 6. Simulation and Analysis

In this part, we propose the simulation and analysis. In Section 6.1, a theoretical analysis has been given for a two base station scenario, link level transmission has been adopted in this part, then theoretical analysis of our proposed method is analyzed [34,35,36]. To make our method credible and practicable, we propose a system level simulation to support the system level performance of our method in Section 6.2. The system level simulation platform supports the multi-users, multi-base stations scenario, which could reflect the performance of our proposed algorithm in a real scenario. References for our simulation platform can be found in [37,38].

### 6.1. Theoretical Analysis

**Figure 2:**
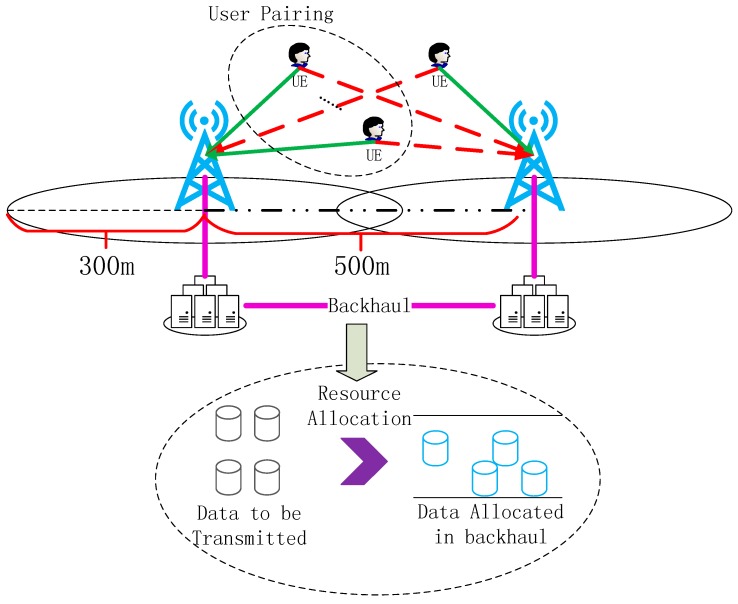
Scenario of the theoretical analysis. Two base stations are connected through the cooperative link and our proposed method is given.

First of all, we evaluate the performance of our algorithm on the MATLAB platform. The distance between two BSs is 500 m. The radius of each cell is 300 m, which is illustrated in Figure 2. In the uplink scenario, users are transmitting signals to both of the base stations and signals are also interference to an adjacent base station. Two base stations are connected using the backhaul link. User pairing is proposed in coordinated demodulation of the received signal and the resource allocation in backhaul link is proposed to make full use of the system. During each timeslot, there are eight users transmitting simultaneously via eight subcarriers in each cell and their positions are randomly generated. Wireless channel taken in our simulation is multipath Rayleigh fading with pass loss exponent α=2.6 and 8 paths with power [1 0 0 2.0053 0 0 0 1.2646] in dB. We have evaluated the sum rate of users in both cells achieved by our algorithm under different backhaul resource.

As the benchmarks, the performance of Random Pairing with Equal Backhaul resource Allocation (RPEB) and Random Pairing with Random Backhaul resource Allocation (RPRB) schemes are also presented. Specifically, both RPEB and RPRB let α be the same as in our proposed algorithm, β is a random mapping; RPRB lets ***r*** be uniformly distributed among all subcarriers; RPRB generates ***r*** randomly. For a given backhaul resource, the achievable sum rate under each scheme is averaged by 100 channel generations.

**Figure 3:**
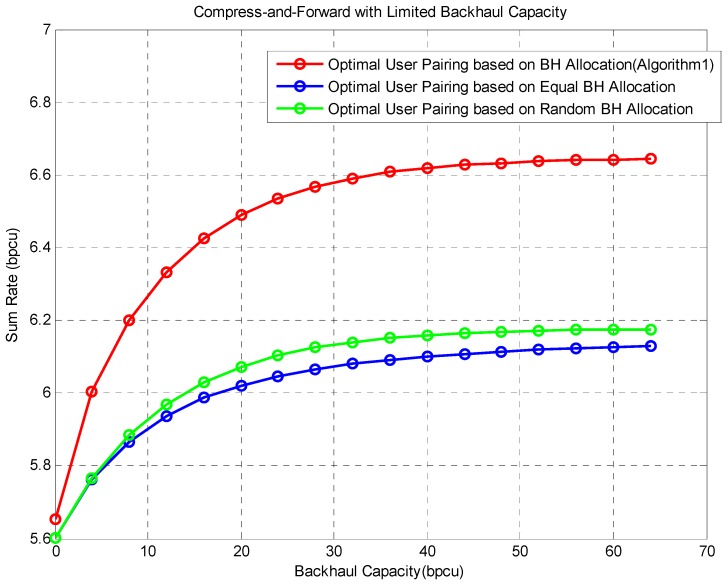
Comparison of different methods. The red curve is our proposed optimal method, the green one is the method using equal backhaul resource allocation and the blue one is the performance of random resource allocation.

The following Figure 3 compares the average sum-rate achieved by different schemes. We can make the following observations:

Our proposed algorithm outperforms two benchmarks by a significant margin. As the backhaul resource grows, this margin also grows. This is because the proposed algorithm has more and more freedom to allocate backhaul resources. Until the backhaul resource is sufficient, this margin stays stable. It verifies that optimal backhaul resource allocation is necessary to obtain better system performance [30].

These three curves have a common tendency. As the backhaul resource grows, the sum rate at first increases approximately linearly, and then it rises slowly, and finally, it enters into a stable stage. This is because in this low backhaul resource region, system capacity is limited by backhaul resources rather than additional information BS B can offer. In other words, the upper cut set bound is dominant. However, at the high backhaul resource region (larger than 60 bpcu as depicted in the figure), the additional information offered by BS B becomes the limiting factor. That is the upper multiple access channel bound is dominant. In this region, the sum rate is saturated.

In addition, when the backhaul resource equals 0, all three curves converge to the same point. This is because the three schemes have the same initialization at this point. In other words α is the same.

### 6.2. System Level Simulation Results

In this part, the system level simulation results have been given. Different from the theoretical analysis, the system level simulation result is close to the real scenario, which is widely used in evaluation of the algorithm. In this section, we propose the scenario with seven base stations, where every base station is divided into three 120 degree sectors. The intersite distance is 500 m for regular transmission.

**Figure 4:**
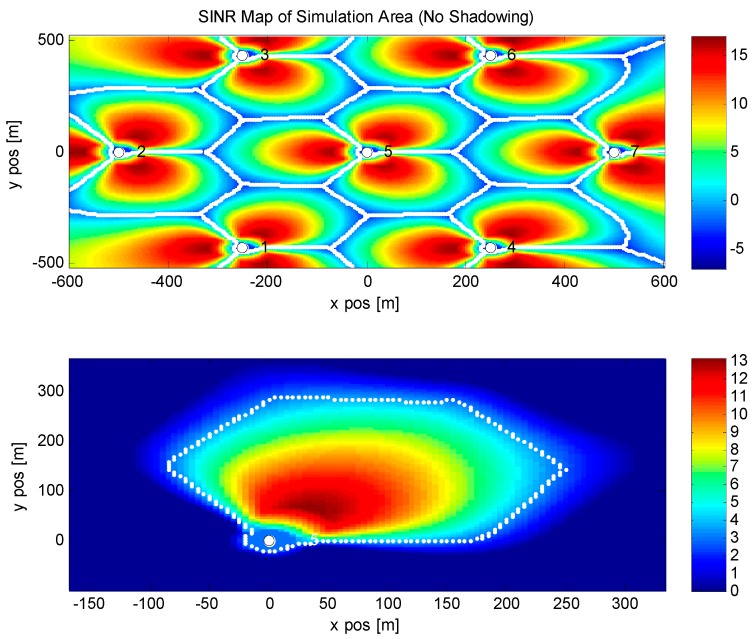
SINR map without shadowing of our simulated area. No shadowing is considered in this figure, so the received SINR is only depending on the distance to the base station from the user. Sectors are quite clear with 120 degree sector angle.

In Table 1, we list all parameters and assumptions for the proposed simulation. The main scenario is based on the 3GPP LTE-Advanced Winner Channel Model used in [33]; the Urban Micro channel scenario is considered, which reflects the fact of a typical load in wireless personal communications. For the LTE-Advanced system without carrier aggregation, 20 MHz bandwidth and 2 GHz carrier frequency are considered, 2 by 2 MIMO mode is also selected, but the transmission mode is simple, and the antennas at the base station side are working separately. As is shown in the following figure, each transmission antenna will send signals to its fixed received antenna only, that means the channel matrix is diagonal. The transmission power of the system is set to 6.3 W for micro or pico base stations. The number of users located in the system is 210 in total. For every base station, there are 30 users on average with the speed of 1 m/s. The maximum cooperative point is 3, in this simulation, as is shown in Figure 4, base station 5,6,7 is a group and base stations 2 and 3, and base stations 1 and 4 are the other two groups. Within each group, base stations are connected using the capacity constrained backhaul link. Cooperative delay is set to a uniform distribution of *U*(5,15). Other parameters are listed in the following table.

**Figure 5:**
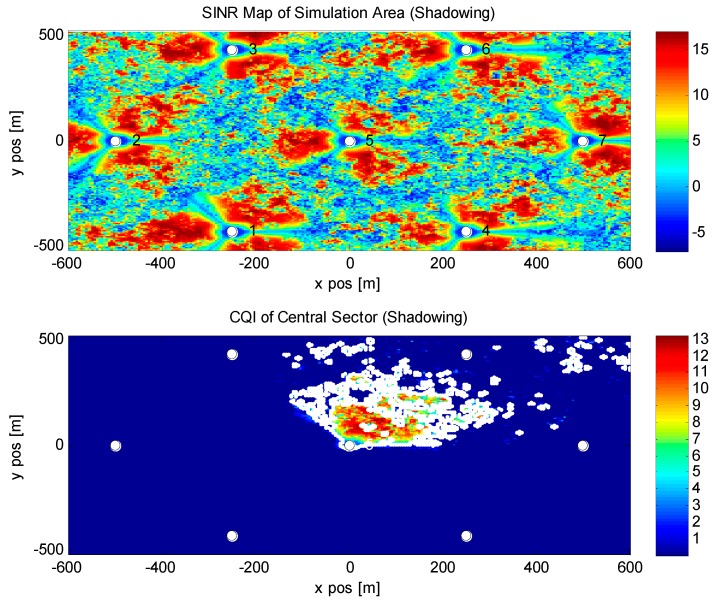
SINR map with shadowing of our simulated area. Shadow fading is considered in this figure, buildings, trees and other loose impediments will affect the radius of the wireless signal.

In Figure 4 and Figure 5, a SINR map of the simulated area is given. We simulate a 7-base stations scenario which is commonly used in LTE-related simulations. Figure 4 is the result of SINR map without shadow fading, the x-axis and the y-axis are the distance in meters, different colors indicate the strength of the received signal. Blue means the received SINR is low and red means the received SINR is high. It is obvious that when the position of a user is close to a base station, the condition of the received signal is perfect and it is suitable for cooperation. When users are located at the edge of the cell, it is difficult to receive satisfactory signals, so the cooperation is not valid at such a position. In Figure 5, shadow fading is considered in the same scenario. Different from the SINR map above, the shadowing map adds the influence of trees, buildings and other sources, which will be the reflectors to the channels. Such scattering will affect the transmission of wireless signals in a line of sight (LOS) scenario, that is why even when a user is close to the base station the performance is poor.

**Figure 6:**
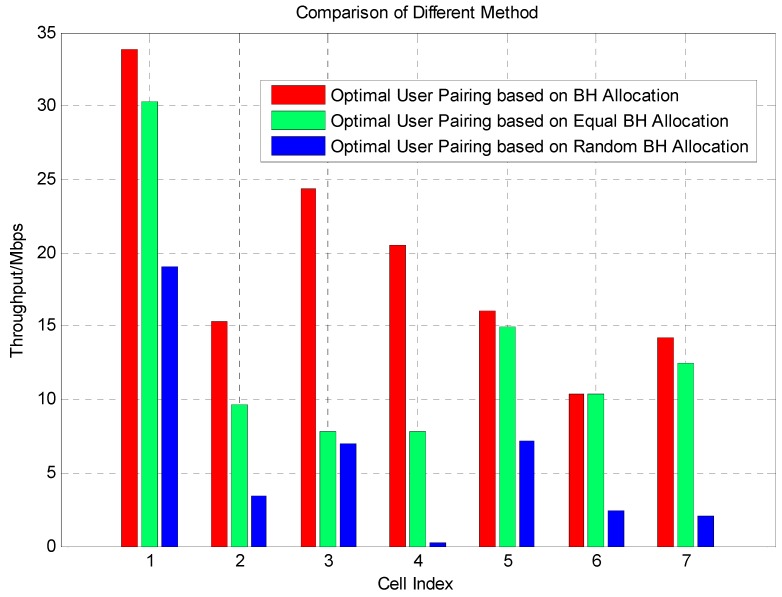
Comparison of different methods for different base stations. Cell index means the index of base stations in our simulated area.

In Figure 6, the statistical result of system average throughput for different base stations is given. The x-axis is the index of different base stations and y-axis is the average throughput in Mbps. Different colors indicate different methods. Red color represents the proposed method using our optimal backhaul resource allocation, green represents the method using equal backhaul resource allocation and blue represents the random allocation. It is obvious that for every base station, the proposed optimal backhaul resource allocation method could increase system capacity significantly compared with other two reference methods. The capacity constraint backhaul will limit the performance of joint processing, but a proper resource allocation scheme will make up the influence of limited backhaul, so from base station 1 to 7, our proposed method gain the maximum performance. The equal BH allocation scheme is the second best method, because the equal allocation is a fair method. Random allocation is the worst one because random allocation is a Monte Carlo method and only reflects the average level of the capacity constraint backhaul. To verify our method in depth, we propose the comparison of SINR *versus* average throughput and block error rate (BLER) to make it clear.

**Figure 7:**
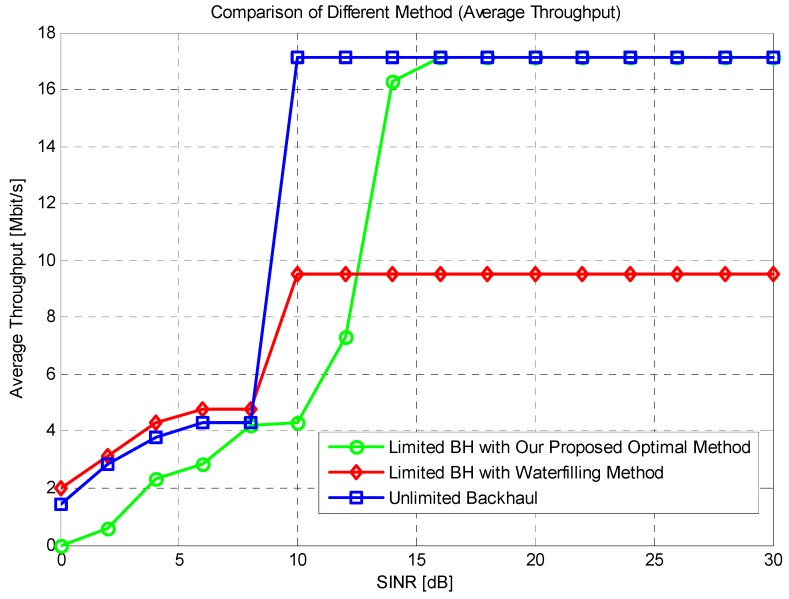
Throughput performance of different SINR.

**Figure 8:**
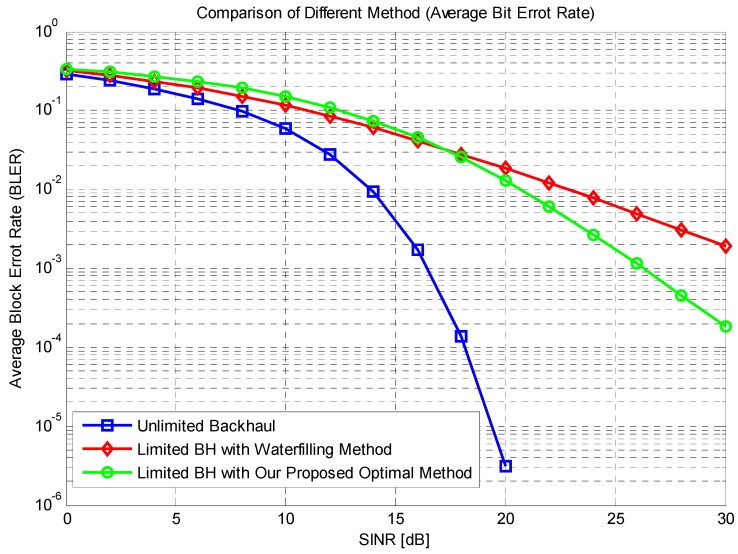
Average BLER of different SINR.

In Figure 7, the result of SINR (Signal to Interference plus Noise Ratio) *versus* average throughput is given. The x-axis is the system SINR in dB and the y-axis is the system average throughput in Mbps. Different colors and legends mean different methods. The blue curve is the performance without the constraint of system backhaul, the red one is the performance using water filling method given in [31] and the green one is our proposed method. First of all, the unlimited backhaul cooperative mode reaches the top when the SINR is 10 dB, and water filling method also reaches its bottleneck of 9.5 Mbps at the same SINR value. It is clear that the capacity constraint backhaul greatly affects the system performance, and there is about 50% performance loss. When adopting our proposed method, the maximum throughput could be achieved through good signal strength, when the SINR is about 16 dB, the system could also reach the top performance; this is because the power makes up for the loss of limited bandwidth, which is also proved in the Shannon formula, but when the SINR is low, our proposed method does not work well, it is because the resource allocation scheme requires a minimum transmission speed as is given in the proof, so in this figure, we can conclude that our proposed method could fix the disadvantage of limited backhaul bandwidth and maintain satisfied performance through our optimal resource allocation in backhaul link. The performance of BLER (Block Error Rate) will be given in Figure 8.

In Figure 8, the result of SINR *versus* average BLER is given. Legends and axis have the same meaning as Figure 7. In this figure, the performance of BLER is proposed. First, with the increasing system SINR, the cooperation with unlimited backhaul has the best performance as expected. Between our proposed method and the water filling method, the difference is not quite clear, and only when the SINR is high, the optimal allocation method performs a little better. This is because the transmission power of the system helps increase the system capacity, so the allocation scheme could work better. To conclude the description of this figure, we can infer that the difference of BLER performance is not quite clear compared with the reference method. Only when the SINR is high, the proposed optimal method performs better.

## 7. Conclusions

In this paper, we discuss the joint resource allocation problem in capacity constrained wireless networks, which will be popular when the transmission nodes and system requirements develop in LTE-related systems or IoT-related systems. The main contributions of this work are:

(1) We have studied the uplink optimal resource allocation with limited backhaul resources in a multicarrier coordinated network. A novel efficient algorithm based on an alternating optimization strategy has been put forward to solve the joint optimization problem of user pairing, subcarrier mapping and backhaul resource allocation. Simulation results show that the proposed algorithm can significantly improve system performance compared with blind schemes. They also illustrate that when CSI from neighbor cell is available, the channel aware backhaul allocation outperforms unaware schemes (random or equal strategy).
(2) We discuss the system performance at a system level in advance. Through the system level simulation, we prove that our proposed method could work well with the currently working systems and the complexity is also acceptable. However, when CSI from a neighboring cell is unavailable, equal backhaul resource allocation is fairer, which will be studied in following work.

## Figures and Tables

**Table 1:** Parameters and Assumptions for the Simulation.

Name	Parameter
Cell Layout	7 Cell/21 Sector
Inter Cell Distance	500 m
Transmission Bandwidth	20 MHz
Antenna Configuration	2 × 2
BS TX Power	6.3 Watt
Max Re-transmission time	4
Carrier Frequency	2 GHz
HARQ Scheme	IR
Channel Model	SCME-Urban Micro
Pathloss	L = 128.1 + 37.6 × log10 (R)
Shadowing Std	4 dB
Noise Power	−107 dBm
Service Type	Full Buffer
Simulation TTIs	2000
User Number	210 total, 30 per Cell
UE Speed	1 m/s
CQI measurement	Ideal
Adaptive Modulation and Coding Scheme (AMC)	QPSK (R ={1/8, 1/7, 1/6, 1/5, 1/4, 1/3, 2/5, 1/2, 3/5, 2/3, 3/4, 4/5}) 16QAM (R = {1/2, 3/5, 2/3, 3/4, 4/5})
Target BLER	0.1
Cooperative Delay	5~15 ms
CoMP Mode	Joint Processing
Backhaul Bandwidth	20 MHz
Max CoMP Point	3
